# Harnessing light energy with molecules

**DOI:** 10.3762/bjoc.22.52

**Published:** 2026-05-04

**Authors:** Grace G D Han, Mogens Brøndsted Nielsen, Hermann A Wegner

**Affiliations:** 1 Department of Chemistry and Biochemistry, University of California, Santa Barbara, CA 93016, USA; 2 Department of Chemistry, University of Copenhagen, Universitetsparken 5, 2100 Copenhagen, Denmark; 3 Institute of Organic Chemistry, Justus Liebig University Giessen, 35392 Giessen, Germany; 4 Center of Materials Research (LaMa/ZfM), Justus Liebig University Giessen, 35392 Giessen, Germany

Photoirradiation is one of the most effective ways of introducing energy into a molecule with high spatiotemporal resolution. From photoexcited states, molecules can relax via various pathways, which essentially converts photon energy to other forms of energy, including chemical and thermal energy. This thematic issue highlights various photochemical processes, for example by which some of the photon energy is stored within the molecule and can be potentially used at a later stage. A strong emphasis is on molecular photoswitches (photochromic molecules) that upon irradiation undergo *cis*/*trans* isomerization, intramolecular photocycloaddition, or an electrocyclic reaction. In addition, several articles describe absorption tuning by structural modifications of the chromophores, aiming for example at moving their absorption from the ultraviolet spectral region to the visible region. This is of importance for efficient exploitation of solar energy, i.e., matching molecular absorption with the solar emission spectrum, or for photodynamic therapies using molecular light harnessing.

So-called molecular solar thermal (MOST) energy storage systems are one type of photoresponsive systems [1–3]. These have attracted increasing interest in the past decade for the storage of solar energy by photoisomerization of a molecular photoswitch, recently coined as a “mostophore” [3]. Such energy storage systems have also been referred to as solar thermal fuels or solar thermal batteries. After light harnessing, photoisomerization generates a metastable compound of higher energy. Thermal back-reaction results in the release of the energy again as heat. This process is preferably induced by an external stimulus or by a catalyst, which enables a controlled energy release when needed. Overall, the process corresponds to a closed cycle of energy uptake and release.

This thematic issue has a strong focus on MOST systems and showcases various approaches of optimization with regard to their light harnessing efficiency, energy storage capacity, and storage time. Structural modifications and studies of the norbornadiene/quadricyclane (NBD/QC) photo-/thermoswitch couple are covered in several articles. In a publication by Kerzig, Ihmels, and co-workers [4], the π-system of monoaryl-substituted NBDs was synthetically extended by acetylenic bridges. In addition, Hebborn, Ihmels, and co-workers [5] show that ^19^F NMR spectroscopy enables unambiguous identification of mono- and bisQC intermediates in the sequential photoreactions of a fluorinated trisNBD-benzene, overcoming the signal overlap that limits conventional ^1^H NMR analysis. Further, the consequences on the absorption properties, photoisomerization, and QC lifetime were studied. In another article by Krappmann and Hirsch [6], the implications of replacing the methylene bridge of the NBD structure by a heteroatom (oxygen or nitrogen) in donor–acceptor, push–pull NBD derivatives are reported. In a computational study, Pawar and co-workers [7] further expanded the NBD structure by elongating the unsaturated bridge with different heteroatoms or functional groups. Azobenzenes, interconverting between *trans* and *cis* isomers, represent another class of photoswitches, which were reviewed as mostophores in a Perspective by Wang, Wu, and co-workers [8].

Besides MOST systems, molecular photoswitches are interesting for a variety of potential applications when incorporated as photoresponsive units in larger systems or smart materials [9]. Photoswitches are, for example, relevant functional units for information storage or molecular electronic devices. They can serve to control ion transport, molecular assembly/disassembly, or the relative movement of units within molecular machines. In a Review, García-López and co-workers [10] describe how the integration of a variety of molecular photoswitches into mechanically interlocked systems, i.e., rotaxanes, can be explored for light-controlled movements, and hence for controlling advanced functions of smart materials and biofunctional systems. In another publication, Leung and co-workers [11] describe how amphiphilic donor–acceptor Stenhouse adducts can be employed in supramolecular nanostructures controlled by visible light, which is of particular interest for the development of biomedical materials. Jones, Evans, and co-workers [12] studied X-ray-induced isomerization within micelles generated from two different photosurfactants (containing either an azobenzene or arylazopyrazole photoswitch), thereby creating guidelines for using small-angle X-ray scattering to study photoresponsive materials of potential applications in solar energy storage, catalysis, or controlled drug delivery. In the article by Venkataramani, Gopakumar, and co-workers [13], it is shown how a fluorinated tripodal N-functionalized arylazo-3,5-dimethylpyrazole derivative, self-assembled on a graphite surface, can be switched between eight states in a tunneling junction at ambient conditions and hence be used as an 8-bit operation unit. Molecular photoswitches are also interesting for temperature sensing applications. Thus, in their publication, Priimagi and co-workers [14] show a strong temperature-dependence of azobenzene protonation in 1,2-dichloroethane.

Systematic tuning of photochromic properties is the focus of several articles in this thematic issue. Kitagawa, Kobatake, and co-workers [15] studied the effect of the substitution position of aryl groups on the thermal back-reaction of azadiarylethene photoswitches. Simeth and co-workers [16] present a study of substituent effects in a large selection of N-acetylated phenylazopyrazole photoswitches. Pittelkow, Antonov, and co-workers [17] present a study on tautomerism and switching in 7-hydroxy-8-(azophenyl)quinoline and similar π-expanded azobenzene compounds. The photoisomerization quantum yield is a key property for characterizing the light harnessing efficiency of a photoswitch. In the article by Volker, Steen, and Crespi [18], a convenient and reliable fiber optic spectroscopic setup for determining this quantum yield is reported and was benchmarked for azobenzene against other methods.

Photoinduced charge separation is a key process in the development of systems for artificial photosynthesis, photovoltaics, and photocatalysis. Kobayashi and co-workers [19] studied in detail the excited-state dynamics of donor–acceptor dyads based on perylene and phenothiazine, in which triphenylamine units and a phenyl spacer were introduced to modulate donor strength and spatial separation. The work underscores the crucial role of excited-state structural relaxation in tuning photoinduced charge separation. In another study, Kerzig and co-workers [20] show how polyaza[7]helicene can function as a singlet-state photoredox catalyst in the sulfonylation/arylation of styrenes and as a triplet sensitizer in energy transfer catalysis.

In addition to the photoredox contribution mentioned above, this thematic issue contains two publications on photochemical reactions beyond photoisomerizations. In the article by Wahl and co-workers [21], ring-opening of photogenerated azetidinols was used as a strategy for the convenient synthesis of aminodioxolanes. Photochemical denitrogenation reactions of bicyclic azoalkanes present a way to prepare strained bicyclic compounds. Further, Gomes and Lopez [22] report a computational study on the mechanism of photodenitrogenation of diazabicyclo[2.2.1]heptenes.

We are grateful to all authors who have contributed to this thematic issue. The contributions show how harnessing of light energy can be finely tuned by structural modifications and used for diverse applications.

Grace G. D. Han, Mogens Brøndsted Nielsen and Hermann A. Wegner

Santa Barbara, Copenhagen and Giessen, April 2026

## Data Availability

Data sharing is not applicable as no new data was generated or analyzed in this study.
